# Varying the ratio of Lys: Met through enhancing methionine supplementation improved milk secretion ability through regulating the mRNA expression in bovine mammary epithelial cells under heat stress

**DOI:** 10.3389/fvets.2024.1393372

**Published:** 2024-06-25

**Authors:** Lin Fu, Yinjie You, Yu Zeng, Qifan Ran, Yan Zhou, Rui Long, Heng Yang, Juncai Chen, Juan J. Loor, Gaofu Wang, Li Zhang, Xianwen Dong

**Affiliations:** ^1^Chongqing Academy of Animal Sciences, Chongqing, China; ^2^College of Veterinary Medicine, Southwest University, Chongqing, China; ^3^College of Animal Science and Technology, Southwest University, Chongqing, China; ^4^Mammalian NutriPhysioGenomics, Department of Animal Sciences and Division of Nutritional Sciences, University of Illinois, Urbana, IL, United States

**Keywords:** heat stress, nutrition requirement, amino acid, milk secretion ability, RNA sequencing

## Abstract

**Introduction:**

The ratio of lysine (Lys) to methionine (Met) with 3.0: 1 is confirmed as the “ideal” profile for milk protein synthesis, but whether this ratio is suitable for milk protein synthesis under HS needs to be further studied.

**Methods:**

To evaluate the molecular mechanism by which HS and Lys to Met ratios affect mammary cell functional capacity, an immortalized bovine mammary epithelial cell line (MAC-T) is incubated with 5 doses of Met while maintaining a constant concentration of Lys. The MAC-T cells was treated for 6 h as follow: Lys: Met 3.0: 1 (control 37°C and IPAA 42°C) or treatments under HS (42°C) with different ratios of Lys: Met at 2.0: 1 (LM20), 2.5: 1 (LM25), 3.5: 1 (LM35) and 4.0: 1 (LM40). RNA sequencing was used to assess transcriptome-wide alterations in mRNA abundance.

**Results:**

The significant difference between control and other groups was observed base on PCA analysis. A total of 2048 differentially expressed genes (DEGs) were identified in the IPAA group relative to the control group. Similarly, 226, 306, 148, 157 DEGs were detected in the LM20, LM25, LM35 and LM40 groups, respectively, relative to the IPAA group. The relative mRNA abundance of *HSPA1A* was upregulated and anti-apoptotic genes (*BCL2L1* and *BCL2*) was down-regulated in the IPAA group, compared to the control group (*p <* 0.05). Compared with the IPAA group, the relative mRNA abundance of anti-apoptotic genes and casein genes (*CSN1S2 and CSN2*) was up-regulated in the LM25 group (*p <* 0.05). The DEGs between LM25 and IPAA groups were associated with the negative regulation of transcription RNA polymerase II promoter in response to stress (GO: 0051085, DEGs of *BAG3*, *DNAJB1*, *HSPA1A*) as well as the mTOR signaling pathway (ko04150, DEGs of *ATP6V1C2*, *WNT11*, *WNT3A*, and *WNT9A*). Several DEGs involved in amino acids metabolism (*AFMID*, *HYKK*, *NOS3*, *RIMKLB*) and glycolysis/gluconeogenesis (*AFMID* and *MGAT5B*) were up-regulated while DEGs involved in lipolysis and beta-oxidation catabolic processes (*ALOX12* and *ALOX12B*) were down-regulated.

**Conclusion:**

These results suggested that increasing Met supply (Lys: Met at 2.5: 1) may help mammary gland cells resist HS-induced cell damage, while possibly maintaining lactation capacity through regulation of gene expression.

## 1 Introduction

In a high temperature and humidity environment, the imbalance between heat accumulation and dissipation in dairy cows can induce heat stress (HS) (1). Once the ambient temperature rises above the threshold and the body is unable to dissipate heat effectively, the cow will be under HS due to the disruption of internal homeostasis (2). Due to the innate self-protection mechanisms of animals, several biological processes are initiated, such as reducing dry matter intake and rumination time, increasing respiratory rate and hormone secretion, to alleviate the negative effects of HS (3). The lactation performance of dairy cows is severely negatively affected by HS, which seriously undermines the economic efficiency of dairy farming.

Mammary gland is the most important organ for milk synthesis and secretion. In addition to the decline in milk production, the protein content of milk also decreases during the hot summer months (4, 5), partly due to the direct negative impact on milk protein synthesis in the mammary gland (6). Milk synthesis and secretion are considered system processes incredibly sensitive to both physiological and environmental factors (7–9). Previous study suggested that inadequate feed intake, changes in postabsorptive metabolism and nutrient partitioning may contribute to discordant changes in mammary protein synthesizing capacity in heat-stressed cows (10). In addition, the apoptosis rate of mammary epithelial cells in dairy cows was increased under HS conditions, and cytoskeletal and cell transport functions were disturbed (11–13). In modern dairy farms, managers adopt a variety of approaches to alleviate or prevent the occurrence of HS in dairy cows, and some feed additives [betaine (14), choline (15), taurine (16) and methionine (17)] seem to be effective in alleviating negative effects of HS.

Methionine (Met) and lysine (Lys) are the most-limiting amino acids in a large range of diets for dairy cows (18). Previous studies indicated that an approximately 3.0: 1 ratio of Lys to Met in dietary metabolic proteins can increase the yield of milk protein to an optimal level (19, 20), which is considered as the “ideal” amino acid profile (IPAA) for milk protein synthesis. However, during HS, the uptake of amino acids (including Met) of dairy cows is altered, resulting in inhibition of the synthesis of milk protein content (21). Increasing the supply of Met in bovine mammary epithelial cells (BMECs) reduced apoptosis and necrosis, decreased lipid peroxidation, and increased the activities of superoxide dismutase, catalase, and glutathione peroxidase, resulting in comprehensive cytoprotective effects under high temperature conditions (17, 22, 23). Thus, whether the 3.0: 1 ratio of Lys: Met is ideal to promote milk protein synthesis under HS conditions and the regulatory mechanisms are not well known.

Our hypothesis was that changing the ratio of Lys: Met by increasing or decreasing Met supplementation could be a way to help mitigate the negative impact of HS on BMECs. To address this hypothesis, an immortalized bovine mammary epithelial cell line (MAC-T) was cultured with different ambient temperature conditions: thermo-neutral (37°C) and HS (42°C), and 5 media contains 175 mM Lys and varying Met concentrations (58 mM, 44 mM, 50 mM, 70 mM, and 87 mM). The RNA sequencing (RNA-Seq) approach was used to identify the molecular mechanisms regulated by changes in Met supplementation.

## 2 Materials and methods

### 2.1 Cell culture and treatments

An immortalized bovine mammary cell line (MAC-T) was chosen as the model. The MAC-T cells were derived from our laboratory, and the cell culture protocol followed our previous similar study with minor modifications (24). Briefly, the thawed bovine MAC-T cells were cultured in 75cm^2^ flasks using an incubator at 37°C and 5% CO_2_ until sufficient cells were obtained for subsequent experiments. The basal medium was prepared with minimum essential medium with Earle’s balanced salts (GE Healthcare Life Sciences, Logan, UT) and fetal bovine serum at a ratio of 9.0: 1, and supplemented with 5 mg/L insulin, 1 mg/L hydrocortisone, 5 mg/L transferrin, 5 μM ascorbic acid, 5 mM sodium acetate, 100 U/mL penicillin, 100 μg/mL streptomycin, 0.25 μg/mL antimycotic, 1 mg/L progesterone, 0.05% lactalbumin, and 0.05% α-lactose. When confluency reached 80–90%, the cells were digested with trypsin–EDTA solution and re-inoculated in 6-well plates. The basal medium was replaced by the lactogenic medium when the cell confluency reached 80–90% again, followed by the plates were incubated overnight at 37°C. The lactogenic medium was changed minimum essential medium with Earle’s balanced salts in the basal medium to high-glucose Dulbecco’s modified Eagle’s medium (Hyclone, GE Healthcare Life Sciences), and supplemented with 1 g/L bovine serum albumin and 2.5 mg/L prolactin. Subsequently, the lactogenic medium was changed to the special lactogenic medium containing different ratios of amino acids (as presented in Table 1), and the cells were further at cultured 37°C or 42°C for 6 h (27). Accordingly, there were 6 treatments as follow: 37°C treatment with Lys: Met 3.0: 1 (control), 42°C treatments with Lys: Met at 2.0: 1 (LM20), 2.5: 1 (LM25), 3.0: 1 (IPAA), 3.5: 1 (LM35) and 4.0: 1 (LM40). After incubation for 6 h, cell samples were collected and stored at −80°C until RNA extraction. The reagents and chemicals were purchased from Sigma-Aldrich (St. Louis, MO) unless otherwise stated.

**Table 1:** Amino acid composition of the lactogenic medium.

Amino acid (μg/mL)	Treatments^a^					
	Control^b^	IPAA^b^	LM40	LM35	LM25	LM20
Lys	175	175	175	175	175	175
Met	58	58	44	50	70	87
Lys/Met	3.0: 1	3.0: 1	4.0: 1	3.5: 1	2.5: 1	2.0: 1
Thr	97	97	97	97	97	97
Phe	93	93	93	93	93	93
His	74	74	74	74	74	74
Val	142	142	142	142	142	142
Ile	121	121	121	121	121	121
Leu	206	206	206	206	206	206
Arg	84	84	84	84	84	84
Trp	16	16	16	16	16	16

a Control and IPAA treatments containing Lys: Met at 3.0, LM40, LM35, LM25 and LM20 treatments containing Lys: Met at 2.0: 1, 2.5: 1, 3.5: 1 and 4.0: 1, respectively.

b The ideal amino acid composition is described as previously described (25, 26).

### 2.2 RNA extraction and RT-qPCR analysis

Total RNA was extracted from MAC-T cells using TRIzol reagent (#15596026, Invitrogen, United States) and RNA quality determined using a NanoDrop 1,000 ND-2000 spectrophotometer (Thermo Scientific, USA). The cDNA synthesis was performed using the PrimeScript RT reagent Kit with gDNA Eraser (Takara Biotechnology, Dalian, China) according to the manufacturer’s instructions. The RT-PCR was performed according to the manufacturer’s instructions using SYBR Premix Ex Taq (Takara Biotechnology, Dalian, China). The cDNA was diluted to 50 ng with RNase-free water and 2 μL of diluted cDNA was combined with the 20 μL reaction mixture. The 20 μL system contained 10 μL of 2 × SYBR Premix Ex Taq (Tli RNsesH Plus), 0.4 μL each of 10 μM forward and reverse primers, 0.4 μL of 50x ROX Reference Dye II and 4.8 μL of RNase-free water. All RT-PCR was performed in a QuantStudio 6 Flex System (Applied Biosystems, Foster City, CA, United States) with the following program: 95°C for 30 s, 40 cycles at 95°C for 5 s, and 60°C for 34 s. The detailed list of primer sequence is presented in Table 2. All primers were commercially manufactured by Sangon Biotech Co., Ltd. (Shanghai, China). Three reference genes (*GADPH*, *UXT*, and *RPS9*) were used to normalize the expression of target genes. The comparative cycle threshold (2^−∆∆Ct^) method was used to determine the mRNA abundance of target genes (28, 29).

**Table 2:** The primer sequences of genes.

Gene		Sequences (5′-3′)	Accession number	Product length
*HSPA5*	Forward	GATCAAGGCAACCGCATCAC	XM_024998380.2	163
	Reverse	GCTGCACGGACGGGTCATT		
*HSP90AB1*	Forward	GCTCAGACGAGGAGGATGATAGT	NM_001079637.1	189
	Reverse	CCAAGTGATCTTCCCAGTCATT		
*HSPB8*	Forward	GGAGGTGTCTGGTAAACACGAAG	NM_001014955.1	184
	Reverse	GCTCTCTCCAAACGGTGAGTAA		
*HSPA1A*	Forward	ACGACGGAGACAAGCCTAAG	NM_203322.3	88
	Reverse	GTCAGCACCATCGACGAGA		
*BCL2L1*	Forward	TGAGCAGGTGTTTTGGACAA	XM_005214498.4	199
	Reverse	CACTGGGGGTTTCCATATCT		
*BCL2*	Forward	TATTCTCAGCGTGTAACTTGTGT	XM_024984176.2	119
	Reverse	TCAGTCTACCTCCTCCGTGA		
*CSN1S1*	Forward	CCCAACAGAAAGAACCTATG	XM_059887320.1	175
	Reverse	CCAATGGGATTAGGGATG		
*CSN2*	Forward	GTGAGGAACAGCAGCAAACA	XM_015471671.3	233
	Reverse	AGGGAAGGGCATTTCTTTGT		
*TSC1*	Forward	TACTGGGCCACGTCGTGAG	XM_059891865.1	102
	Reverse	CGTCGGTGTCCATCTTGAGAC		
*TSC2*	Forward	GCAGCAGGATCCAGACCTCT	XM_059881501.1	112
	Reverse	GTCTCTGTGAGCTCCAGGTGG		
*RHEB*	Forward	GCTAAGATGCCGCAGTCCA	NM_001031764.2	75
	Reverse	CGTCAACGAGGATTTCCCC		
*mTOR*	Forward	CTTCTTCCGTTCCATCTC	XM_002694043.7	116
	Reverse	CTTCCACTAAGGCTTCATT		
*S6K1*	Forward	TGGAACAATAGAATACAT	NM_205816.1	167
	Reverse	GTTTACATTTGAGGATTT		
*EIF4EBP1*	Forward	GGAGTGTCGGAACTCACCTG	NM_001077893.2	162
	Reverse	AACTGTGACTCTTCACCGCC		
*eIF4E*	Forward	AGGGAGGGTATACAAGGAAAGGTT	NM_174310.3	101
	Reverse	TTTTAGTGGTGGAGCCGCTC		
*eEF2K*	Forward	TCTCTGTCCTCAATCAAG	NM_175813.2	110
	Reverse	GGTCTCATCTGTATCTGT		
*eEF2*	Forward	GAGATCCAGTGTCCAGAA	NM_001075121.1	147
	Reverse	GAAGCCAAAGGACTCATT		
*RPS9*	Forward	CCTCGACCAAGAGCTGAAG	NM_001101152.2	64
	Reverse	CCTCCAGACCTCACGTTTGTTC		
*GAPDH*	Forward	TGGAAAGGCCATCACCATCT	XM_001034034.2	53
	Reverse	CCCACTTGATGTTGGCAG		
*UXT*	Forward	TGTGGCCCTTGGATATGGTT	XM_001037471.2	101
	Reverse	GGTTGTCGCTGAGCTCTGTG		

HSPA5, Heat shock protein 5; Hsp90AB1, Heat shock protein 90 kDa alpha class B member 1; HSPB8, Heat shock protein beta-8; HspA1A, Heat shock 70 kDa protein 1A; BCL2L1, B-cell lymphoma-2 apoptosis regulator like 1; BCL2, B-cell lymphoma-2 apoptosis regulator; CSN1S1, ɑS1-casein; CSN2, ɑS2-casein; β-casein; TSC1; TSC2, Tuberous sclerosis complex 2; RHEB, GTP-binding protein Rheb; mTOR, Mammalian Target of Rapamycin; EIF4EBP1, Eukaryotic translation initiation factor 4E binding protein 1; eIF4E, Eukaryotic translation initiation factor 4E; eEF2K, Eukaryotic elongation factor 2 kinase; eEF2, Elongation factor 2; RPS9, 40S ribosomal protein S9; GAPDH, Glyceraldehyde-3-phosphate dehydrogenase; UXT, Ubiquitously expressed transcript protein.

### 2.3 RNA sequencing

Total RNA was extracted using Trizol reagent (Invitrogen, Carlsbad, CA, United States) according to the manufacturer’s protocols. RNA quality was assessed on an Agilent 2,100 Bioanalyzer (Agilent Technologies, Palo Alto, CA, United States) and checked using RNase free agarose gel electrophoresis. After total RNA was extracted, eukaryotic mRNA was enriched by Oligo (dT) beads, while prokaryotic mRNA was enriched by removing rRNA by Ribo-ZeroTM Magnetic Kit (Epicentre, Madison, WI, USA). Then the enriched mRNA was fragmented into short fragments using fragmentation buffer and reverse-transcribed into cDNA with random primers. Second-strand cDNA was synthesized with DNA polymerase I, RNase H, and dNTP. Then, the cDNA fragments were purified with QiaQuick PCR extraction kit (Qiagen, Venlo, The Netherlands), end repaired, A base added, and ligated to Illumina sequencing adapters. The ligation products were size selected by agarose gel electrophoresis, PCR amplified, and sequenced using Illumina Novaseq6000 by Gene Denovo Biotechnology Co., Ltd. (Guangzhou, China). OD260/OD280 values of all samples were ≥ 1.9, and RNA Integrity Number (RIN) values were ≥ 8.0. The cDNA library was constructed using 3 μg total RNA for each sample. Before the library was constructed, the ribosomal RNA was removed by Epicentre Ribo-zero™ rRNA removal kit (Epicentre, United States), and the total RNA removed by rRNA was cleaned by precipitation with ethanol. The NEB Next®Ultra™ Directional RNA Library Prep Kit for Illumina® (NEB, USA) was then used for library construction using RNA with the rRNA removed. Library sequencing was performed using Illumina HiSeq4000 at Guangzhou Gidio Biotechnology Co., Ltd. The short-read alignment tool Bowtie2 (30) (version 2.2.8, https://bowtie-bio.sourceforge.net/bowtie2/index.shtml) was used for mapping reads to the ribosomal RNA (rRNA) database. An index of the reference genome was built and paired-end clean reads mapped to the reference genome using HISAT 2.2.4 (31) with “-rna-strandness RF” and other parameters set as default. The mapped reads for each sample were assembled with StringTie v1.3.1 (32, 33) in a reference-based approach. Principal component analysis (PCA) was performed with R package gmodels (http://www.rproject.org/). RNA differential expression analysis between two different groups was assessed via DESeq2 (34, 35). The genes/transcripts with a false discovery rate (FDR) below 0.05 and absolute fold change ≥1.5 were considered as differentially expressed genes (DEGs). The DEGs were annotated by Gene ontology (GO) functional enrichment and Kyoto Encyclopedia of Genes and Genomes (KEGG) pathway enrichment using the R programming language (3.5 version, http://www.r-project.org/), based on the hypergeometric distribution.

### 2.4 Statistical analysis

The mRNA abundance data of each gene were log_2_ transformed to obtain a normal distribution before statistical analysis. The statistical analysis was performed using the MIXED model in SAS (version 9.3; SAS Institute Inc., Cary, NC, United States) with Lys to Met ratios as the main fixed effect and individual cell culture well as random effect. Treatment means were generated using the LSMEANS option and separated when they were significant with the PDIFF option. Statistical significance was considered at *p <* 0.05.

## 3 Results

### 3.1 Heat shock response

**Figure 1:**
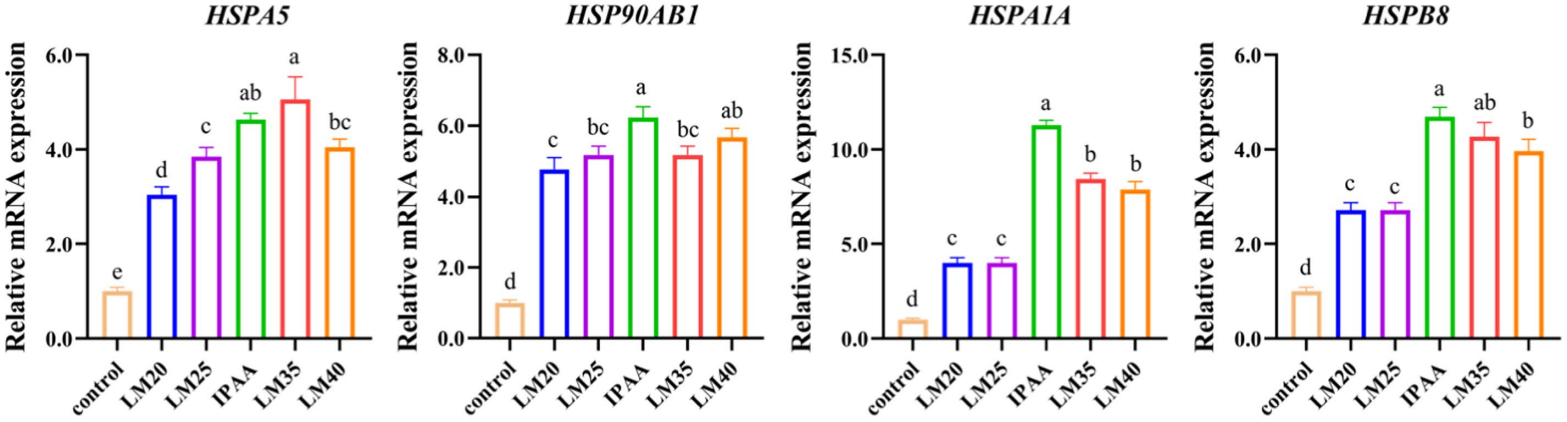
The relative mRNA expression of heat shock response genes in MAC-T cells with the different treatments. Control (37°C) and IPAA (42°C) treatments containing Lys: Met at 3.0, LM40, LM35, LM25 and LM20 containing Lys: Met at 2.0: 1, 2.5: 1, 3.5: 1 and 4.0: 1, respectively. Asterisks indicated significant differences between different groups: * *p <* 0.05.

The effects of HS on the mRNA expression of heat shock response genes are shown in Figure 1. HS up-regulated the gene expression of *HSPA5*, *HSP90AB1*, *HspA1A*, and *HSPB8* (*p <* 0.05). However, under HS, the gene expression of *HSPA5*, *HSP90AB1*, *HSPA1A* and *HSPB8* was down-regulated in the LM25 and LM20 group (*p <* 0.05), the gene expression of *HSP90AB1* and *HSPA1A* was down-regulated in the LM35 group (*p <* 0.05), the gene expression of *HSPA1A* and *HSP8* was down-regulated in the LM35 group (*p <* 0.05), compared to the IPAA group.

### 3.2 Abundant of apoptosis-related genes

**Figure 2:**
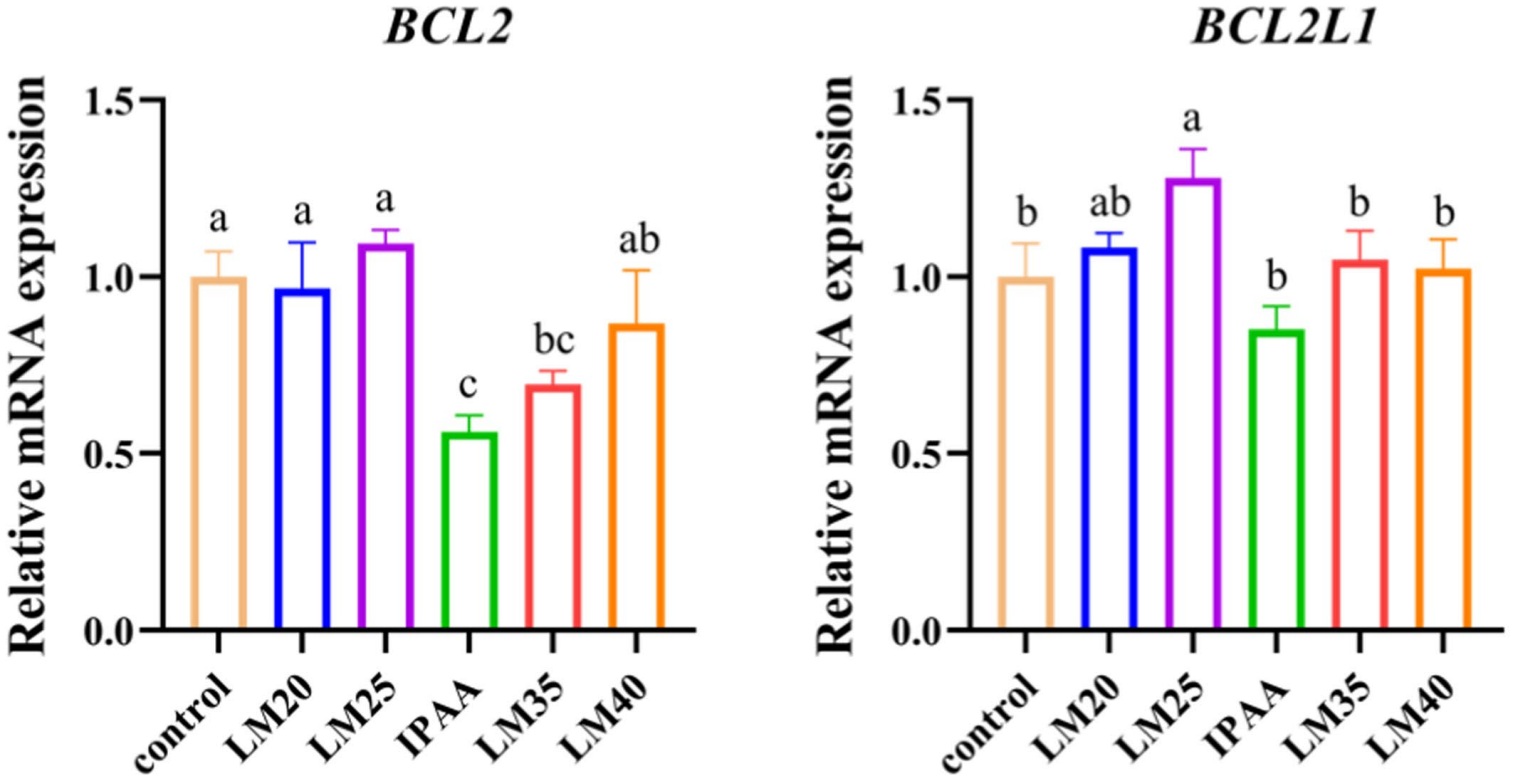
The relative mRNA expression of apoptosis-related genes in MAC-T cells with the different treatments. Control (37°C) and IPAA (42°C) treatments containing Lys: Met at 3.0: 1, LM40, LM35, LM25 and LM20 containing Lys: Met at 2.0: 1, 2.5: 1, 3.5: 1 and 4.0: 1, respectively. Asterisks indicated significant differences between different groups: * *p* < 0.05.

Compared with the control group, the gene expression of *BCL2* was down-regulated in the IPAA group (*p <* 0.05, Figure 2). Under HS, compared with the IPAA group, the gene expression of *BCL2* was up-regulated in the LM20, LM25 and LM40 groups (*p <* 0.05), the gene expression of *BCL2L1* was up-regulated in the LM25 group (*p <* 0.05).

### 3.3 mRNA expression of casein genes

**Figure 3:**
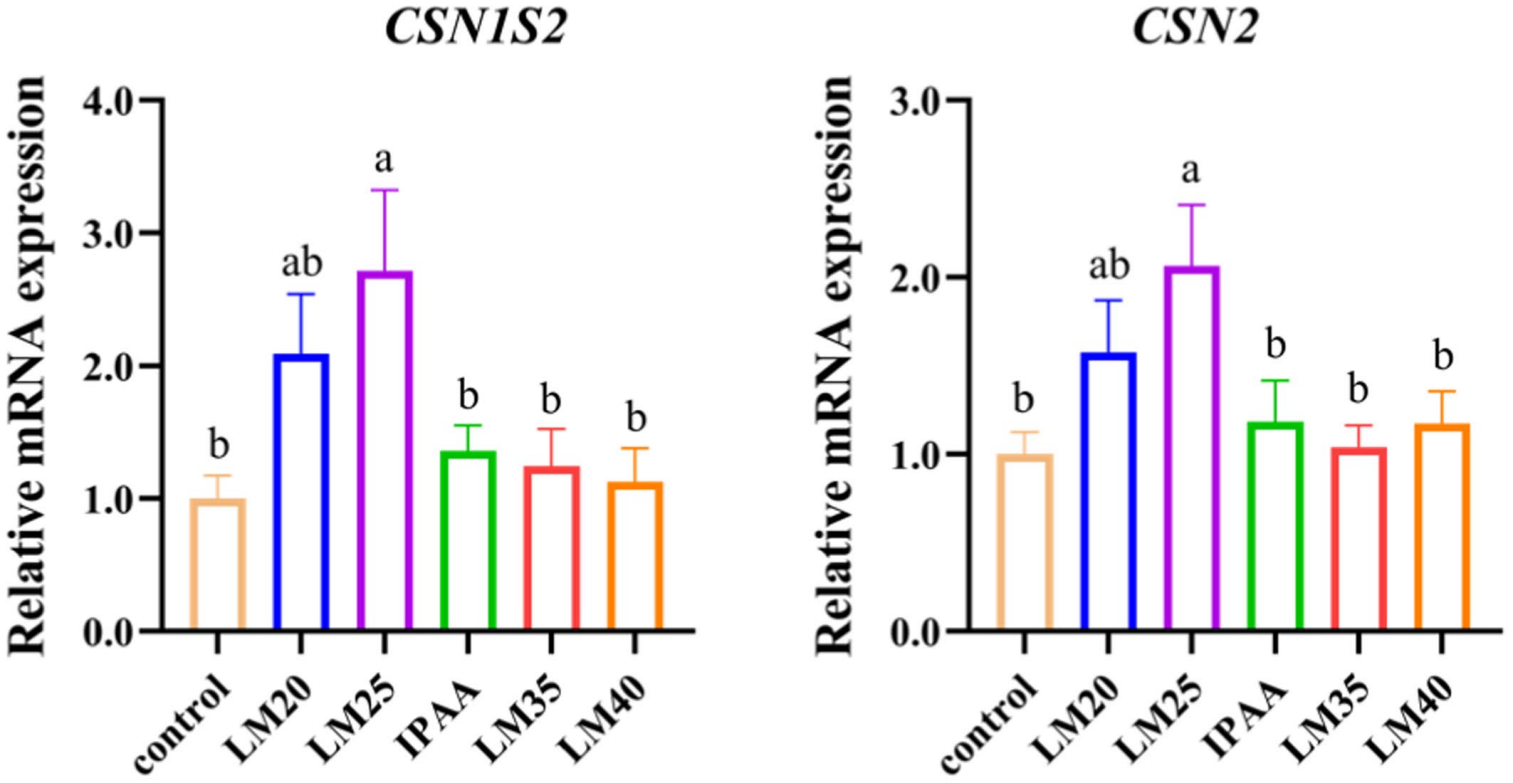
The relative mRNA expression of casein genes in MAC-T cells with the different treatments. Control (37°C) and IPAA (42°C) treatments containing Lys: Met at 3.0: 1, LM40, LM35, LM25 and LM20 containing Lys: Met at 2.0: 1, 2.5: 1, 3.5: 1 and 4.0: 1, respectively. Asterisks indicated significant differences between different groups: * *p* < 0.05.

The expression of *CSN1S2* and *CSN2* in the LM25 group was up-regulated compared with the control group (*p <* 0.05, Figure 3).

### 3.4 RNA sequencing results

**Figure 4:**
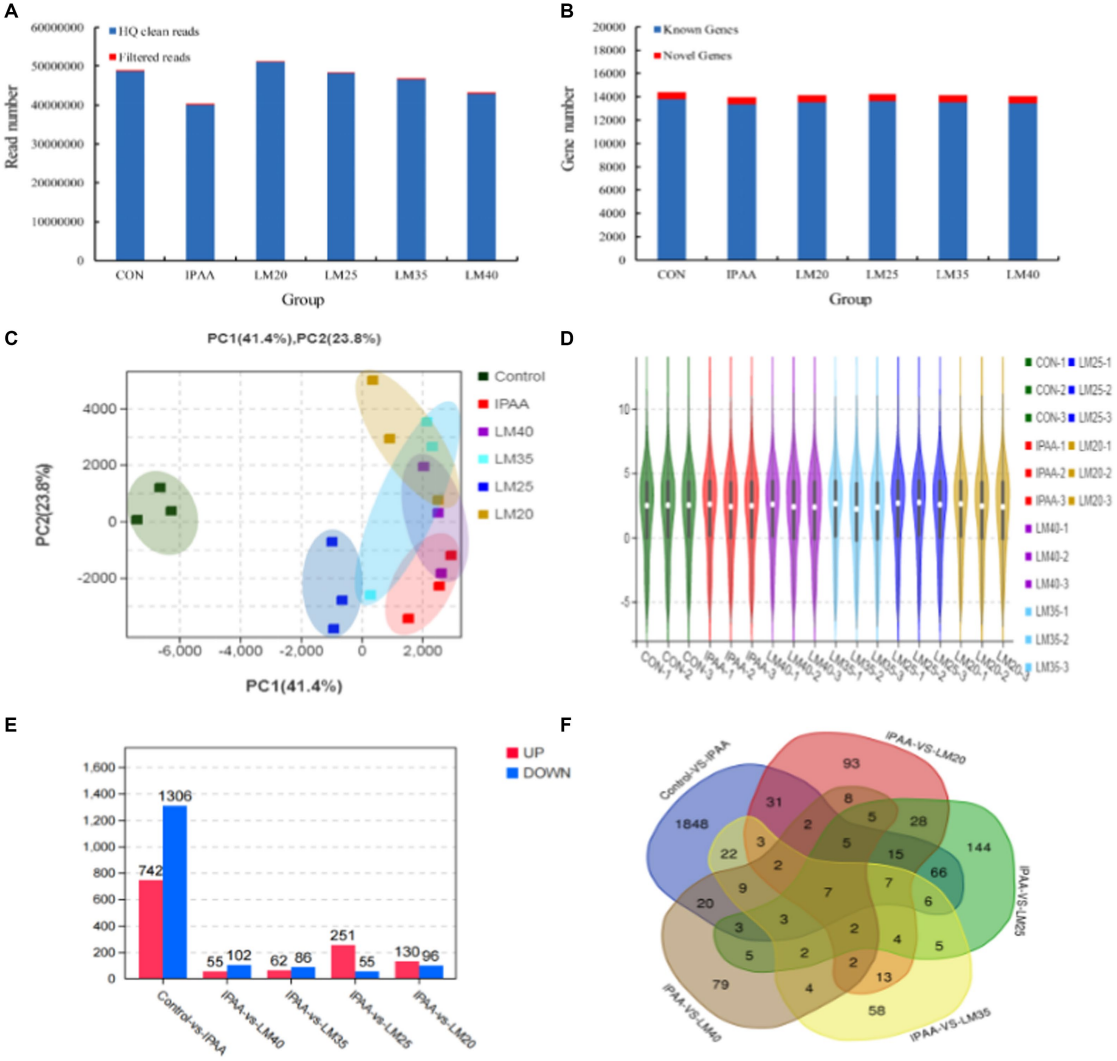
Regulation of gene transcription expression in MAC-T cells with the different treatments. Control (37°C) and IPAA (42°C) treatments containing Lys: Met at 3.0: 1, LM40, LM35, LM25 and LM20 containing Lys: Met at 2.0: 1, 2.5: 1, 3.5: 1 and 4.0: 1, respectively. Read number obtained by RNA sequencing **(A)**; genes number identified by RNA sequencing **(B)**; PCA analysis **(C)**; sample cluster analysis **(D)**; number of differentially expressed genes **(E)**; Venn diagram **(F)**.

A total of 3.8–5.1 million raw sequencing reads were generated in each group. The high-quality (HQ) clean reads obtained accounted for more than 99% of all the raw reads (Figure 4A) and were mapped to the bovine reference genome (*Bos Taurus*, assembly ARS-UCD1.2). The mean mapping ratio was greater than 96% in each group. A total of 13,773, 13,390, 13,552, 13,623, 13,528 and 13,456 known genes and 591, 591, 585, 592, 593 and 592 new genes were identified in the control, IPAA, LM20, LM25, LM35 and LM40 groups, respectively (Figure 4B). The cumulative variance contribution rate (65.2%, PC1 + PC2) of the principal component analysis (PCA) for the gene expression profiles was lower than the standard of 85% (Figure 4C). The principal component analysis illustrated that a significant difference existed between the control and other groups under HS. At the same time, except for the IPAA group, the three repeats in each HS groups tended to cluster closely, indicating that adding different concentrations of Met resulted in a high similarity in the overall expression levels of core genes during HS (Figure 4C). The sample clustering analyses further confirmed the PCA results (Figure 4D).

To investigate the regulatory mechanisms for the effects of Met on lactation performance, gene function analysis was performed. Genes with a *p*-value <0.05 and an absolute value of log_2_ fold-change (|log_2_ FC|) > 2 were considered as DEGs. A total of 2048 DEGs were screened resulting in 742 upregulated and 1,306 downregulated in the IPAA group relative to the control group. A total of 306 DEGs with 251 upregulated and 55 downregulated were in the LM25 group relative to the IPAA group, and 130, 62, 55 upregulated and 96, 86, 102 downregulated were detected in the LM20, LM35 and LM40 group, respectively, relative to the IPAA group (Figure 4E).

Increasing Met supplementation significantly affected the expression of 28 DEGs under HS (Figure 4F; Supplementary Table S1). These included *CCN1* (cellular communication network factor 1, *CCN1*) and *ZNF182* (zinc finger protein 182, *ZNF182*), which play a role in cell proliferation, differentiation, and apoptosis. In addition, *FGF2 1* (fibroblast growth factor 21, FGF21) and *AFMID* (arylformamidase, AFMID) function as metabolic regulators. In contrast, decreasing Met supplementation significantly affected the expression of 4 DEGs under HS (Figure 4F; Supplementary Table S1) including *SELPLG* (selectin P ligand, SELPLG), *NR1H4* (nuclear receptor subfamily 1, group H, member 4, NR1H4).

**Figure 5:**
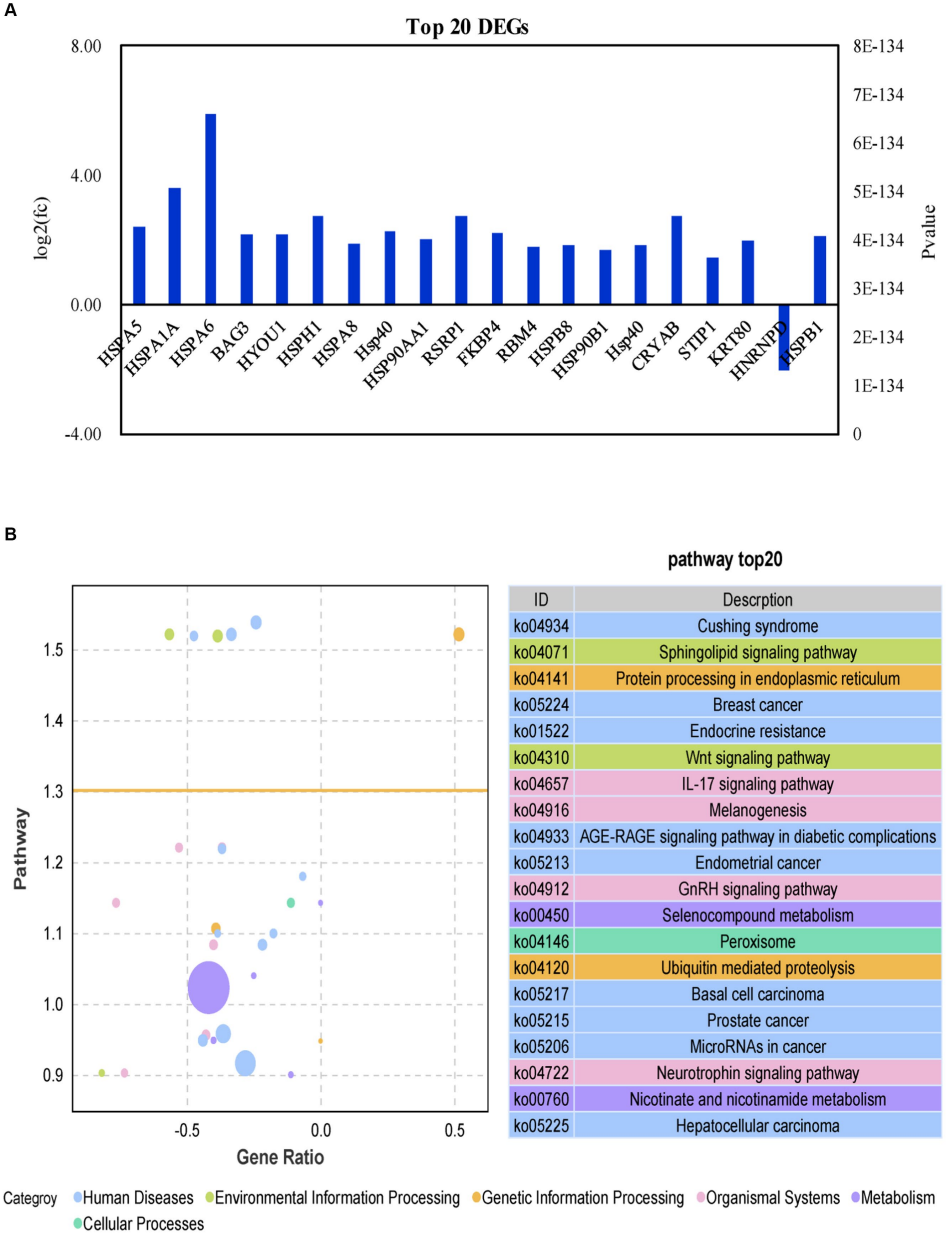
Analysis of differential genes function in MAC-T cells with the the different treatments. Control (37°C) and IPAA (42°C) treatments containing Lys: Met at 3.0: 1, LM40, LM35, LM25 and LM20 containing Lys: Met at 2.0: 1, 2.5: 1, 3.5: 1 and 4.0: 1, respectively. Top 20 differential genes **(A)**; KEGG analysis **(B)**.

The top 10 DEGs encoding heat shock proteins (*HSPA5*, *HSPA1A*, *HSPA6*, *HSPH1*, *HSPA8*, *DNAJA4*, *HYOU1* and *HSP90AA1*) were highly up-regulated during HS in the IPAA group relative to the control group (Figure 5A). *BAG3* (BAG cochaperone 3, *BAG3*), associated with apoptosis, was also upregulated. For more in-depth biological function information, KEGG pathway annotation analysis was performed using the DEGs list with the assistance of KEGG database. The enriched pathways with *p* value <0.05 are reported in Figure 5B. Eight pathways in the Control-*vs*-IPAA condition had a close relationship to the autoimmune disorders including cushing syndrome, breast cancer, endocrine resistance, AGE-RAGE signaling pathway in diabetic complications, endometrial cancer, basal cell carcinoma, prostate cancer, and MicroRNAs in cancer. Some of the pathways are associated with cellular responses by HS including sphingolipid signaling pathway, protein processing in the endoplasmic reticulum, Wnt signaling pathway, IL-17 signaling pathway, GnRH signaling pathway, ubiquitin mediated and proteolysis, and peroxisome. Two pathways are related to metabolism including Selenocompound metabolism and nicotinate and nicotinamide metabolism. Nearly all these top-affected pathways are related to immune and heat shock response. The responsiveness of bovine MAC-T cells to HS in this study, clearly suggested its suitability as a model to understand the modulation of cow mammary gland expression signatures in response to HS.

**Figure 6:**
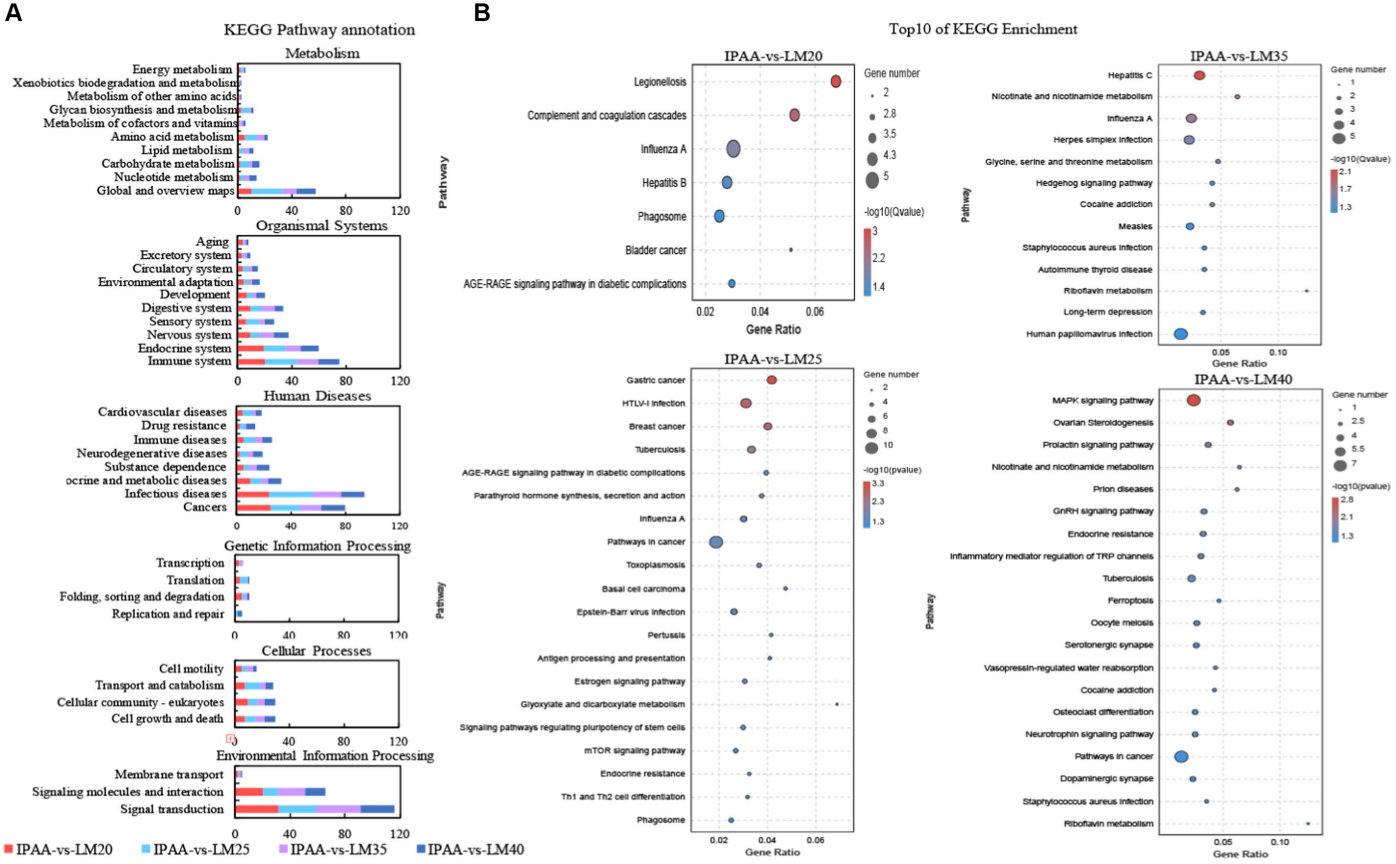
KEGG analysis of differential genes in MAC-T cells with the different treatments. Control (37°C) and IPAA (42°C) treatments containing Lys: Met at 3.0: 1, LM40, LM35, LM25 and LM20 containing Lys: Met at 2.0: 1, 2.5: 1, 3.5: 1 and 4.0: 1, respectively. KEGG Pathway annotation **(A)**; Top10 of KEGG Enrichment **(B)**.

The KEGG pathway annotation analysis revealed that DEGs were mainly involved in amino acid metabolism, immune system, infectious diseases, and signal transduction (Figure 6A). In addition, most DEGs (*DNAJB1*, *EEF1A2*, *EGR1*, *EGR2*, *FGF21*, *HSPA1A*, *MAPK12*, and *WNT3A*) among the four groups were all significantly enriched in immunology-related pathways involved in the heat stock response (Figure 6B; Supplementary Table S2). In addition, the DEGs of the LM25 group were also significantly enriched in glyoxylate and dicarboxylate metabolism and mTOR signaling pathway (Figure 6B). The DEGs of the LM35 group were significantly enriched in nicotinate and nicotinamide metabolism, glycine, serine and threonine metabolism (Figure 6B). The DEGs of the LM40 group were markedly enriched in the MAPK signaling pathway and nicotinate and nicotinamide metabolism (Figure 6B).

The effects of Met supplementation on mRNA abundance of milk fat, lactose, and mTOR signaling pathway genes is reported in Table 3. Compared with the IPAA group, the mRNA abundance of *LPL*, *ACACA*, *SCD*, *FADS1*, *GPAM*, *PGM2*, *LPIN1*, *SPTLC1*, *SPTLC2*, *INSIG1*, *INSIG2* and *PPARG* was up-regulated in the LM25 group, as well as the lactose synthesis genes of *UGP2*, *B4GALT1* and *GALE*. In addition, an upregulation in the expression of *EIF4E*, *EEF2K*, and *RHEB*, and a downregulation in expression of *EIF4EBP1* and *eEF2* were also detected in the LM25 group. It was observed that the Met concentration at 70 mM in the LM25 group resulted in a higher expression of genes coding for milk fat, lactose and mTOR signaling-responsive genes.

**Table 3:** The mRNA abundance (log_2_FC) of milk fat, lactose, and mTOR singal pathway genes in MAC-T cells with the different treatments.

Genes	Treatments^a^					
	Control	IPAA	LM40	LM35	LM25	LM20
Milk fat synthesis						
*LPL*	0.08	0.13	0.08	0.07	0.11	0.06
*PGM2*	7.5	9.46	7.74	9.32	14.3	11.12
*LPIN1*	13.76	9.74	8.15	9.26	12.06	7.01
*SPTLC1*	12.99	5.77	4.88	5.63	8.55	5.2
*SPTLC2*	84.8	105.43	90.59	99.73	124.85	103.11
*GPAM*	1.05	1.5	1.69	1.64	2.71	2.02
*ACACA*	18.94	16.82	15.32	15.44	19.07	14.86
*FADS1*	2.87	1.83	1.43	1.57	1.85	1.37
*SCD*	188.39	194.17	190.56	187.26	252.07	186.42
*VLDLR*	18.56	13.04	11.36	9.98	14.61	12.72
*ACSL1*	2.83	2.61	2.88	2.47	3.94	2.95
*PPARG*	0.21	0.12	0.11	0.1	0.14	0.1
*INSIG1*	39.26	49.14	48.12	51.63	67.74	53.84
*INSIG2*	5.84	7.02	5.92	6.71	7.3	6.48
Lactose synthesis						
*GALE*	47.7	34.81	43.52	40.25	41.14	34.87
*HK2*	25.15	36.68	35.21	32.37	46.92	33.43
*UGP2*	24.11	23.29	18.92	22.54	27.13	22.77
*HK1*	118.84	107.39	96.25	98.39	99.04	95.36
*B4GALT1*	63.55	72.54	66.96	62.87	73.74	69.21
Milk protein synthesis						
*mTOR*	16.69	17.28	17.77	16.59	20.43	17.18
*EIF4E*	43.62	41.85	39.69	42.28	48.57	44.75
*EEF2K*	24.36	25.57	24.23	21.76	30.53	21.72
*EIF4EBP1*	83.64	80.81	86	84.09	69.81	92.61
*RHEB*	28.56	31.15	30.99	34.3	35.25	34.36
*EEF2*	1183.62	1116.51	1033.31	1007.04	971.53	1003.23
*TSC2*	24.33	11.02	9.48	8.98	9.05	7.27

a Control and IPAA treatments containing Lys: Met at 3.0, LM40, LM35, LM25 and LM20 treatments containing Lys: Met at 2.0: 1, 2.5: 1, 3.5: 1 and 4.0: 1, respectively.

**Figure 7:**
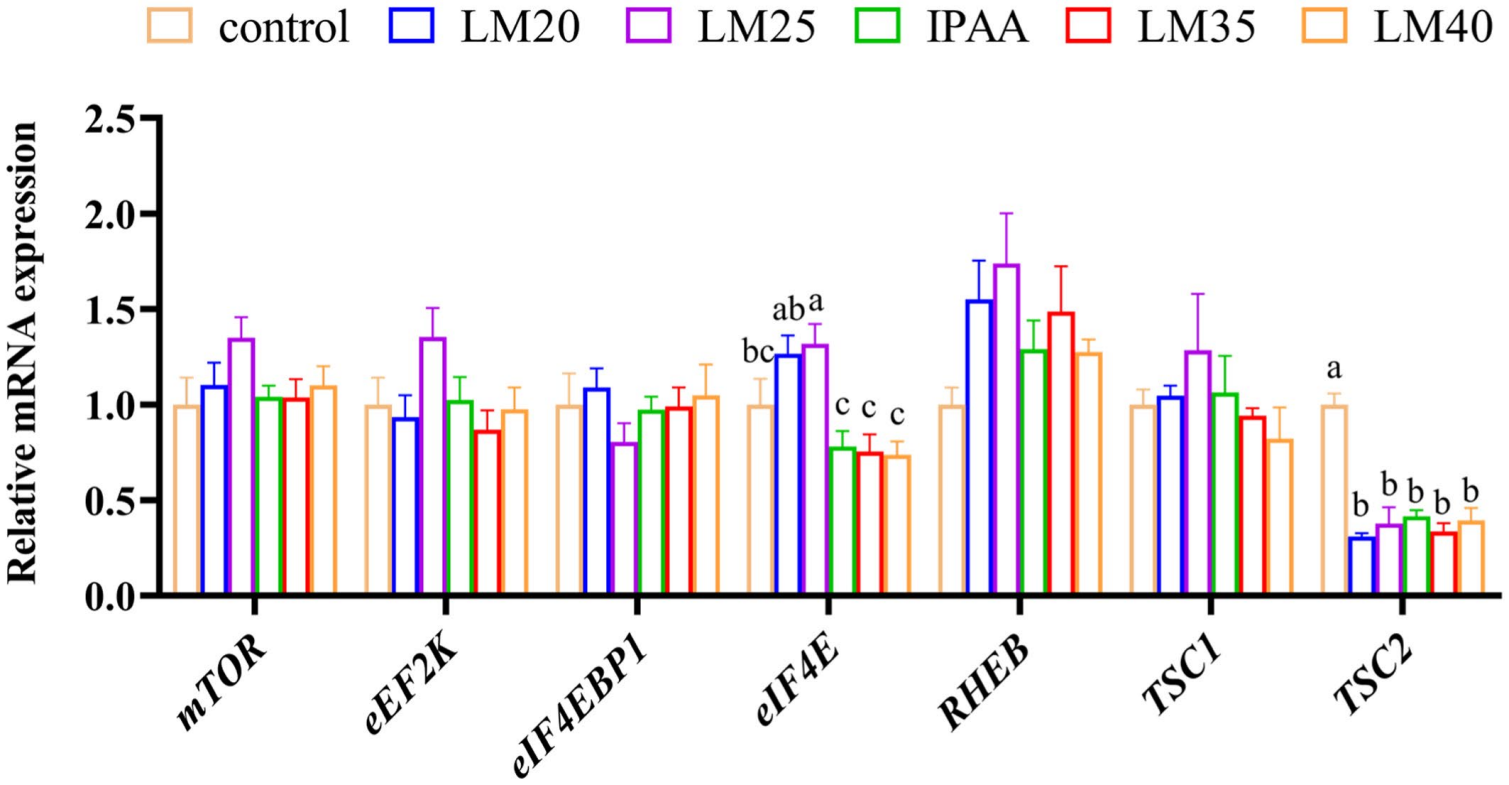
The relative mRNA expression of mTOR signaling pathway related genes in MAC-T cells with the different treatments. Control (37°C) and IPAA (42°C) treatments containing Lys: Met at 3.0: 1, LM40, LM35, LM25 and LM20 containing Lys: Met at 2.0: 1, 2.5: 1, 3.5: 1 and 4.0: 1, respectively. Asterisks indicated significant differences between different groups: * *p* < 0.05.

As presented in Figure 7, the RT-qPCR results were consistent with the RNA-Seq data. The relative mRNA expression of *eIF4E* in the LM25 group was significantly higher than that in the IPAA, LM35 and LM40 groups (*p <* 0.05). Compared with the control group, the relative expression of *TSC2* in all other groups was significantly down-regulated (*p <* 0.05).

## 4 Discussion

The heat shock protein (HSPs) family members constitute a group of chaperone proteins that exhibit rapid up-regulation in response to HS, thereby ensuring cellular homeostasis through the regulation of protein folding and maturation within cells (36). In accordance with previous findings (37–39), the expression of genes encoding Hsps (*HSPA5*, *HSPA1A*, *HSPA6*, *HSPH1*, *HSPA8*, *DNAJA4*, *HYOU1* and *HSP90AA1*) was significantly up-regulated in MAC-T cells upon exposure to HS in this study. It has been demonstrated that the 70 kDa heat shock protein (HSP70) is a reliable biomarker for monitoring changes in body temperature in mammals. These proteins contribute significantly to the heat tolerance of cells by up-regulating protein expression to help restore homeostasis in heat-exposed cells (40–43). The supplementation of Met (70 mg/L) decreased the protein level of HSP70 compared with the control group (60 mg/L Met) (44). Improving the supply of Met has also been reported to prevent heat-induced oxidative stress and significantly reduce mortality of BMECs *in vitro* (23). In this study, the relative mRNA abundance of the genes encoding HSP70 (*HSPA5, HSPA1A*, *HSPA6* and *HSPA8*) was down-regulated with the increase of Met addition under HS, indicating that enhancing Met supply has a potential role in increasing the tolerance of MAC-T cells to heat.

Apoptosis is the ultimate outcome of mammalian cells undergoing sustained HS. In this process, cell death occurs due to the programmed control of genes and the stepwise activation of the apoptotic pathway. B-cell lymphoma 2 (BCL-2)-associated athanogene 3 (BAG3) protein is a co-chaperone of HSP70, acts by binding to the ATPase domain to help the chaperone release ADP and nucleotide cycle (45), and responds to HS with elevated expression. It also has the binding site for BCL-2, an intrinsic (mitochondria-dependent) pathway leading to apoptosis, as well as activation of macrophage phagocytosis through co-infection with HSPs (46). The level of the anti-apoptotic BCL-2 family protein Bcl-xL decreased with the knockdown of BAG3 (47). In this study, compared to the control group, the gene expression of *BAG3* was higher in the IPAA group, while the mRNA level of the anti-apoptotic gene (*BCL2*) was lower in the IPAA group. Moreover, the mRNA level of the anti-apoptotic genes (*BCL2* and *BCL2L1*) was higher in the LM25 group than that in the IPAA group. This result is consistent with previous reports (17), which may be related to the fact that Met effectively triggers the anti-apoptotic response in cells during HS (48). Thus, enhancing Met supply up-regulated the expression of anti-apoptotic genes in MAC-T cells, which may help alleviate heat-induced apoptosis.

There could be a direct link between the decline in milk production and the down-regulation of gene expression associated with milk protein synthesis caused by hyperthermia (49). The ratio of Lys to Met has been demonstrated to alter the expression of casein genes in BMECs (20, 50, 51). In the current study, the mRNA level of casein genes (*CSN1S1* and *CSN2*) was the highest in the LM25 group. There was a dose-dependent relationship between the synthesis of milk fat and the supply of Met, and the secretion of triglycerides and lipid droplets was greatest in BMECs at a dose of 0.6 mM (52). Similarly, in this study, the transcriptional abundance of genes related to *de novo* synthesis of fatty acids (*ACACA*, *SCD* and *FADS1*), triacylglycerol synthesis (*GPAM*, *PGM2* and *LPIN1*), sphingolipid synthesis (*SPTLC1* and *SPTLC2*), and transcription regulation (*INSIG1*, *INSIG2* and *PPARG*) were up-regulated in the LM25 group compared to the IPAA group. Following mammary cell uptake, glucose is converted to uridine diphosphate (UDP-) glucose and UDP-galactose in the cytoplasm under the action of UDP glucose pyrophosphorylase (UGP2) and galactose epimerase (GALE). Finally, one molecule of UDP-galactose and one molecule of glucose are combined by β-1,4-galactosyltransferase 1 (B4GALT1) in the Golgi apparatus to produce lactose (53). The transcript abundance of *UGP2*, *B4GALT1* and *GALE* was upregulated in the LM25 group compared to the IPAA group. The differences in the expression levels of these genes (related to casein, milk fat and lactose synthesis) indicated that the ratio of Lys to Met at 2.5: 1 may be more conducive to the synthesis of milk components in mammary cells under HS conditions.

There is growing evidence that the mammalian target of rapamycin (mTOR) signaling pathway is the central node of the amino acid regulatory pathway that controls the synthesis of milk protein, milk fat and lactose (54–57). Previous studies have demonstrated that increased availability of Met and arginine (54), tryptophan (58) and Lys (59) could affect milk protein synthesis by changing the mTOR signaling pathway. Additionally, our earlier study has also indicated that modifications in the intracellular metabolism of glutamate, arginine and proline, alanine, aspartate, and tryptophan can provide sufficient substrates and energy for milk protein synthesis during HS (24). Thus, the upregulation of genes involved in amino acid metabolism (*AFMID*, *HYKK*, *NOS3*, and *RIMKLB*) in the LM25 group confirmed the biological correlation between Met supply and the mTOR signaling pathway compared to the IPAA group during HS. The mTOR signaling pathway also regulates the metabolism of lipids and carbohydrates by up-regulating the expression of related genes to control enzyme synthesis (60). Compared with the control group, the transcriptional abundance of *AFMID* and *MGAT5B* (involved in glyoxylate and dicarboxylate metabolism as well as mannose type O-glycan biosynthesis) was up-regulated in the LM25 group, indicating that the increased supply of Met may also regulate carbohydrate and lipid metabolism in MAC-T cells through the mTOR signaling pathway.

Taken together, the data from this study indicated that an increased supply of Met (Lys: Met at 2.5: 1) had the ability to attenuate cellular damage (e.g., apoptosis) during HS. In addition, increasing the supply of Met may help increase the synthesis of casein, milk fat, and lactose in mammary cells, in part by altering the expression of genes involved in intracellular metabolism of amino acids, lipids, and carbohydrates, as well as the mTOR signaling pathway. The limitation of this study is that only the MAC-T cell model was used to investigate the increase in Met supply to mitigate the negative effects of HS on milk secretion ability, rather than *in vivo*. Thus, more studies should be conducted on dairy cows to validate the results of this study.

## 5 Conclusion

Heat stress causes changes at the level of gene transcription in MAC-T cells. These changes are partially reversed by the addition of Met supply (ratio of Lys to Met of 2.5:1). The potential mechanism is related to the mRNA expression regulation of HSPs, anti-apoptosis and milk component synthesis genes explored by whole transcription sequencing technology. The findings of this study raise the possibility supplementation with Met might have a positive effect on mammary cells during HS.

## Data availability statement

The original contributions presented in the study are publicly available. The data presented in the study are deposited in the NCBI repository, accession number PRJNA1119483. https://www.ncbi.nlm.nih.gov/sra/?term=PRJNA1119483.

## Ethics statement

Ethical approval was not required for the studies on animals in accordance with the local legislation and institutional requirements because only commercially available established cell lines were used.

## Author contributions

LF: Formal analysis, Methodology, Software, Writing – original draft, Writing – review & editing. YY: Software, Validation, Writing – review & editing. YuZ: Methodology, Resources, Writing – review & editing. QR: Project administration, Supervision, Writing – review & editing. YaZ: Project administration, Supervision, Writing – review & editing. RL: Methodology, Resources, Writing – review & editing. HY: Investigation, Methodology, Software, Writing – review & editing. JC: Data curation, Software, Writing – review & editing. JL: Investigation, Validation, Writing – review & editing. GW: Funding acquisition, Writing – review & editing. LZ: Data curation, Formal analysis, Funding acquisition, Investigation, Methodology, Writing – review & editing. XD: Data curation, Investigation, Methodology, Software, Writing – review & editing.
