# Supplementary material for: Varying the ratio of Lys: Met through enhancing methionine supplementation improved milk secretion ability through regulating the mRNA expression in bovine mammary epithelial cells under heat stress
Source: Front Vet Sci. 2024 Jun 25;11:1393372. doi: 10.3389/fvets.2024.1393372 (PMC11231434; doi:10.3389/fvets.2024.1393372)
Supplement: Supplementary file 1 [file Table_1.doc]

**Supplementary Table 1.** Venn analysis of differentially expressed genes in MAC-T bovine mammary epithelial cell with the ideal AA ratio (Lys:Met 2.9:1; control 37℃, IPAA 42℃) or treatments under heat stress containing Lys:Met at 2.5:1 (LM25), 2.0:1 (LM20), 3.0:1 (LM30) and 4.0:1 (LM40).

| Names | total | ID | Symbol |
| --- | --- | --- | --- |
| IPAA-VS-LM20 & IPAA-VS-LM25 & IPAA-VS-LM35 & IPAA-VS-LM40 | 2 | ENSBTAG00000012436 | HES7 |
|  |  | MSTRG.8006 | - |
| IPAA-VS-LM20 & IPAA-VS-LM25 | 28 | ENSBTAG00000048985 | - |
|  |  | ENSBTAG00000020647 | RASL11B |
|  |  | ENSBTAG00000009844 | CCN1 |
|  |  | ENSBTAG00000011624 | FGF21 |
|  |  | ENSBTAG00000018673 | TRIM17 |
|  |  | ENSBTAG00000044047 | SKIDA1 |
|  |  | ENSBTAG00000007125 | AFMID |
|  |  | ENSBTAG00000046774 | ZNF182 |
|  |  | ENSBTAG00000021685 | EEF1A2 |
|  |  | ENSBTAG00000012522 | ZNF283 |
|  |  | ENSBTAG00000050281 | ZNF23 |
|  |  | ENSBTAG00000037965 | - |
|  |  | ENSBTAG00000009876 | C4BPA |
|  |  | ENSBTAG00000009430 | MYRIP |
|  |  | ENSBTAG00000048341 | - |
|  |  | ENSBTAG00000053638 | - |
|  |  | ENSBTAG00000051039 | - |
|  |  | ENSBTAG00000049610 | ZNF234 |
|  |  | ENSBTAG00000010344 | SLC16A14 |
|  |  | ENSBTAG00000046409 | EGR2 |
|  |  | ENSBTAG00000014738 | HOXA11 |
|  |  | ENSBTAG00000015988 | MYH11 |
|  |  | ENSBTAG00000051842 | - |
|  |  | ENSBTAG00000001395 | C1H21orf91 |
|  |  | ENSBTAG00000012389 | - |
|  |  | ENSBTAG00000014599 | LRRC66 |
|  |  | ENSBTAG00000053227 | - |
|  |  | ENSBTAG00000022372 | - |
| IPAA-VS-LM35 & IPAA-VS-LM40 | 4 | ENSBTAG00000031851 | SELPLG |
|  |  | MSTRG.7797 | - |
|  |  | ENSBTAG00000013596 | NR1H4 |
|  |  | ENSBTAG00000023186 | - |

**Supplementary Table 2.** Differential expressed genes of the KEGG pathways in MAC-T bovine mammary epithelial cell with the ideal AA ratio (Lys:Met 2.9:1, IPAA) or treatments under heat stress containing Lys:Met at 2.5:1 (LM25), 2.0:1 (LM20), 3.0:1 (LM30) and 4.0:1 (LM40).

|  | KEGG_A_class | KEGG_B_class | Pathway | Pvalue | Pathway ID | Genes |
| --- | --- | --- | --- | --- | --- | --- |
| IPAA-vs-LM20 | Human Diseases | Infectious diseases | Legionellosis | 0.00 | ko05134 | HSPA1A, HSPA8, EEF1A2, CXCL8 |
|  | Organismal Systems | Immune system | Complement and coagulation cascades | 0.00 | ko04610 | F2, F3, C4BPA, SERPINA5 |
|  | Human Diseases | Infectious diseases | Influenza A | 0.01 | ko05164 | DNAJB1, HSPA1A, HSPA8, CXCL8, IFNA14 |
|  | Human Diseases | Infectious diseases | Hepatitis B | 0.02 | ko05161 | EGR2, EGR3, CXCL8, IFNA14 |
|  | Cellular Processes | Transport and catabolism | Phagosome | 0.03 | ko04145 | ATP6V1E2, TUBB1, FCGR2A, TUBA8 |
|  | Human Diseases | Cancers | Bladder cancer | 0.04 | ko05219 | RPS6KA5, CXCL8 |
|  | Human Diseases | Endocrine and metabolic diseases | AGE-RAGE signaling pathway in diabetic complications | 0.04 | ko04933 | F3, EGR1, CXCL8 |
| IPAA-vs-LM25 | Human Diseases | Cancers | Gastric cancer | 0.00 | ko05226 | CTNNA3, BCL2, WNT11, FGF21, WNT3A, WNT9A |
|  | Human Diseases | Infectious diseases | HTLV-I infection | 0.00 | ko05166 | SPI1, EGR2, WNT11, MYB, EGR1, WNT3A, WNT9A |
|  | Human Diseases | Cancers | Breast cancer | 0.00 | ko05224 | WNT11, FGF21, WNT3A, WNT9A, ESR2 |
|  | Human Diseases | Infectious diseases | Tuberculosis | 0.00 | ko05152 | BCL2, CTSS, TLR6, MAPK12 |
|  | Human Diseases | Endocrine and metabolic diseases | AGE-RAGE signaling pathway in diabetic complications | 0.01 | ko04933 | BCL2, EGR1, NOS3, MAPK12 |
|  | Organismal Systems | Endocrine system | Parathyroid hormone synthesis, secretion and action | 0.01 | ko04928 | PDE4D, GATA3, BCL2, EGR1 |
|  | Human Diseases | Infectious diseases | Influenza A | 0.01 | ko05164 | DNAJB1, HSPA1A, MX1, MAPK12 |
|  | Human Diseases | Cancers | Pathways in cancer | 0.01 | ko05200 | SPI1, CTNNA3, BCL2, WNT11, FGF21, WNT3A, WNT9A, NKX3-1 |
|  | Human Diseases | Infectious diseases | Toxoplasmosis | 0.01 | ko05145 | BCL2, HSPA1A, MAPK12 |
|  | Human Diseases | Cancers | Basal cell carcinoma | 0.02 | ko05217 | WNT11, WNT3A, WNT9A |
|  | Human Diseases | Infectious diseases | Epstein-Barr virus infection | 0.02 | ko05169 | SPI1, BCL2, ENTPD1, HSPA1A, MAPK12 |
|  | Human Diseases | Infectious diseases | Pertussis | 0.02 | ko05133 | IRF8, C4BPA, MAPK12 |
|  | Organismal Systems | Immune system | Antigen processing and presentation | 0.02 | ko04612 | CTSS, HSPA1A |
|  | Organismal Systems | Endocrine system | Estrogen signaling pathway | 0.02 | ko04915 | BCL2, HSPA1A, NOS3, ESR2 |
|  | Metabolism | Carbohydrate metabolism | Glyoxylate and dicarboxylate metabolism | 0.03 | ko00630 | HYI, AFMID , |
|  | Cellular Processes | Cellular community - eukaryotes | Signaling pathways regulating pluripotency of stem cells | 0.03 | ko04550 | WNT11, WNT3A, WNT9A, MAPK12 |
|  | Environmental Information Processing | Signal transduction | mTOR signaling pathway | 0.04 | ko04150 | ATP6V1C2, WNT11, WNT3A, WNT9A |
|  | Human Diseases | Drug resistance | Endocrine resistance | 0.04 | ko01522 | BCL2, ESR2, MAPK12 |
|  | Organismal Systems | Immune system | Th1 and Th2 cell differentiation | 0.05 | ko04658 | GATA3, MAPK12 |
|  | Cellular Processes | Transport and catabolism | Phagosome | 0.05 | ko04145 | ATP6V1C2, CTSS, TLR6 |
| IPAA-vs-LM35 | Human Diseases | Infectious diseases | Hepatitis C | 0.01 | ko05160 | OAS1X, OAS1Z, IFIT1, IFNA14 |
|  | Metabolism | Metabolism of cofactors and vitamins | Nicotinate and nicotinamide metabolism | 0.02 | ko00760 | NT5M, ENPP3 |
|  | Human Diseases | Infectious diseases | Influenza A | 0.02 | ko05164 | RSAD2, OAS1X, OAS1Z, IFNA14 |
|  | Human Diseases | Infectious diseases | Herpes simplex infection | 0.02 | ko05168 | OAS1X, OAS1Z, IFIT1, IFNA14 |
|  | Metabolism | Amino acid metabolism | Glycine, serine and threonine metabolism | 0.03 | ko00260 | PGAM2, GNMT |
|  | Environmental Information Processing | Signal transduction | Hedgehog signaling pathway | 0.03 | ko04340 | EVC2, BOC |
|  | Human Diseases | Substance dependence | Cocaine addiction | 0.03 | ko05030 | CREB3L4, GRIN3B |
|  | Human Diseases | Infectious diseases | Measles | 0.05 | ko05162 | OAS1X, OAS1Z, IFNA14 |
|  | Human Diseases | Infectious diseases | Staphylococcus aureus infection | 0.05 | ko05150 | SELPLG, FCGR2A |
|  | Human Diseases | Immune diseases | Autoimmune thyroid disease | 0.05 | ko05320 | CD80, IFNA14 |
|  | Metabolism | Metabolism of cofactors and vitamins | Riboflavin metabolism | 0.05 | ko00740 | ENPP3 |
|  | Organismal Systems | Nervous system | Long-term depression | 0.05 | ko04730 | RYR1, GNAZ |
| IPAA-vs-LM40 | Environmental Information Processing | Signal transduction | MAPK signaling pathway | 0.00 | ko04010 | CACNA1A, FGFR4, BDNF, PRKACB, MAPK12, MAPK8IP2 |
|  | Organismal Systems | Endocrine system | Ovarian Steroidogenesis | 0.00 | ko04913 | LHCGR, CYP17A1, PRKACB |
|  | Organismal Systems | Endocrine system | Prolactin signaling pathway | 0.01 | ko04917 | LHCGR, CYP17A1, MAPK12 |
|  | Metabolism | Metabolism of cofactors and vitamins | Nicotinate and nicotinamide metabolism | 0.01 | ko00760 | NT5M, ENPP3 |
|  | Human Diseases | Neurodegenerative diseases | Prion diseases | 0.02 | ko05020 | PRKACB, EGR1 |
|  | Organismal Systems | Endocrine system | GnRH signaling pathway | 0.02 | ko04912 | PRKACB, EGR1, MAPK12 |
|  | Human Diseases | Drug resistance | Endocrine resistance | 0.02 | ko01522 | DLL4, PRKACB, MAPK12 |
|  | Organismal Systems | Sensory system | Inflammatory mediator regulation of TRP channels | 0.02 | ko04750 | ALOX12, PRKACB, MAPK12 |
|  | Human Diseases | Infectious diseases | Tuberculosis | 0.02 | ko05152 | CD74, FCGR2A, MAPK12, IFNA14 |
|  | Cellular Processes | Cell growth and death | Ferroptosis | 0.03 | ko04216 | MAP1LC3A, SLC7A11 |
|  | Cellular Processes | Cell growth and death | Oocyte meiosis | 0.03 | ko04114 | REC8, PRKACB, MAPK12 |
|  | Organismal Systems | Nervous system | Serotonergic synapse | 0.03 | ko04726 | CACNA1A, ALOX12, PRKACB |
|  | Organismal Systems | Excretory system | Vasopressin-regulated water reabsorption | 0.03 | ko04962 | ARHGDIG, PRKACB |
|  | Human Diseases | Substance dependence | Cocaine addiction | 0.03 | ko05030 | BDNF, PRKACB |
|  | Organismal Systems | Development | Osteoclast differentiation | 0.03 | ko04380 | SPI1, FCGR2A, MAPK12 |
|  | Organismal Systems | Nervous system | Neurotrophin signaling pathway | 0.03 | ko04722 | ARHGDIG, BDNF, MAPK12 |
|  | Human Diseases | Cancers | Pathways in cancer | 0.04 | ko05200 | SPI1, DLL4, FRAT2, FGFR4, PRKACB, IFNA14 |
|  | Organismal Systems | Nervous system | Dopaminergic synapse | 0.04 | ko04728 | CACNA1A, PRKACB, MAPK12 |
|  | Human Diseases | Infectious diseases | Staphylococcus aureus infection | 0.04 | ko05150 | SELPLG, FCGR2A |
|  | Metabolism | Metabolism of cofactors and vitamins | Riboflavin metabolism | 0.05 | ko00740 | ENPP3 |
